# Microbiology of airway disease in a cohort of patients with Cystic Fibrosis

**DOI:** 10.1186/1471-2334-6-4

**Published:** 2006-01-11

**Authors:** Antonietta Lambiase, Valeria Raia, Mariassunta Del Pezzo, Angela Sepe, Vincenzo Carnovale, Fabio Rossano

**Affiliations:** 1 Department of Biology and Pathology Cellular and Molecular "Luigi Califano"; 2 Regional Cystic Fibrosis Center, University of Naples "Federico II", Italy

## Abstract

**Background:**

Recent reports document an increasing incidence of new Gram-negative pathogens such as *Stenotrophomonas maltophilia *and *Alcaligenes xylosoxidans *isolated from patients with Cystic Fibrosis, along with an increase in common Gram-negative pathogens such as *Pseudomonas aeruginosa *and *Burkholderia cepacia complex*. Furthermore, the increase in multidrug-resistance of such organisms makes the therapeutic management of these patients more problematic. Therefore, careful isolation and identification, and accurate studies of susceptibility to antibiotics are critical for predicting the spread of strains, improving therapeutic measures and facilitating our understanding of the epidemiology of emerging pathogens. The first aim of this study was to determine the incidence and the prevalence of colonization by Gram-negative organisms isolated from respiratory samples of Cystic Fibrosis patients in the Regional Referral Cystic Fibrosis Centre of Naples; the second was to evaluate the spectrum of multidrug-resistance of these organisms.

**Methods:**

Patients (n = 300) attending the Regional Cystic Fibrosis Unit were enrolled in this study over 3 years. Sputum was processed for microscopic tests and culture. An automated system, Phoenix (Becton Dickinson, Sparks, Maryland, USA), was used for phenotypic identification of all strains; the API 20 NE identification system (bioMérieux, Marcy l'Etoile, France) was used when the identification with the Phoenix system was inaccurate. A PCR-RFLP method was used to characterize the organisms in the *Burkholderia cepacia complex*. A chemosusceptibility test on microbroth dilutions (Phoenix) was used. Primary outcomes such as FEV1 were correlate with different pathogens.

**Results:**

During the period of study, 40% of patients was infected by *Pseudomonas aeruginosa*, 7% by *Burkholderia cepacia complex*, 11% by *Stenotrophomonas maltophilia *and 7% by *Alcaligenes xylosoxidans*. Of the strains isolated, 460 were multidrug-resistant. Multiresistant were *Pseudomonas aeruginosa *and *Burkholderia cepacia complex*.

**Conclusion:**

The results confirm previously reported data; in particular, they show an increase the isolation of non-fermentative Gram-negative bacteria in Cystic Fibrosis patients. They also demonstrate increased resistance to antibiotics. Beta-lactams are rarely effective, with exception of ceftazidime, which is the most efficacious agent against multiresistant strains. Aminoglycosides and quinolones are poorly efficacious.

## Background

Cystic Fibrosis (CF) is the most common life-shortening autosomal recessive disease in the white population and afflicts over 30.000 persons in the United States [1]. While the genetic defect in CF is known, the relationship between the abnormal CFTR protein and chronic pulmonary disease has recently become more clear [2]. A hallmark of CF is chronic respiratory infection which may start very early in the life of these patients. Since the early description of CF, pulmonary infection has been recognized as having the greatest role in morbidity and mortality leading to premature death in 90% of patients. The underlying basic defect in CF, the restriction of infection to the lung and the chronic nature of infection characterize the vicious cycle of inflammation. *Pseudomonas aeruginosa *(PA) has been the most common pathogen in the lower airways of CF patients during the last 3 decades. With the improved survival, new emergent pathogens in the CF lung as *Burkholderia cepacia complex *(Bcc), *Stenotrophomonas maltophilia *(SM) and *Alcaligenes xylosoxidans *(AX) have been detected in the last years [3-6]. While it is recognized that CF patients with Bcc infection can have a worsening of lung function, the relationship between SM and clinical outcome remains to be debated [7]. It is possible to prevent or delay the onset of chronic infections in most patients with CF by eliminating cross-infections and by early aggressive antibiotic treatment of the positive sputum culture. Because of the insidious nature of some of the lower respiratory tract infections, frequent bacteriological investigations of sputum or tracheal secretions are necessary in order to apply hygienic measures in the care centers, segregating the infected and the non-infected patients in different wards as well as to prevent or postpone chronic pulmonary infections [8,9]. This is the reason why a continuous survey of microbiological data is mandatory in order to identify most common pathogens in each single centre in terms of annual incidence and prevalence related to primary clinical outcome. The first aim of our study was to evaluate the incidence and prevalence of Gram-negative infection in a CF cohort study.

Recently it has been reported an increasing antibiotic resistance against the most commonly used antibiotics in the CF patients, above all related to emergence of hypermutable bacteria. This implies difficulties on therapeutic approach [10,11]. Moreover current evidence does not provide a clear relationship between clinical impact and multidrug-resistance.

The second aim of our study was to evaluate a three-year trend of multidrug-resistant strains isolated in our Centre and if this contributes to a greater morbidity in these patients.

## Methods

### Study design

Data were collected by a retrospective analysis of database case records of 300 patients (140 males, 160 females; mean age 16.21 years; range 0.5–50 years) attending regularly the Regional Referral CF Centre of Naples between January 2002 and December 2004. Patients over 6 years of age had at least one lung function evaluation as measured by forced expiratory volume in 1 sec (FEV1) during each year of observation at clinic visit and/or hospital discharge expressed of a mean value of three years survey; oxygen saturation measurement was noted for each visit. Regular measure of weight, height, use of pancreatic enzymes, number of CF related hospital admissions, number of courses of intravenous antibiotics were obtained over the observation period. For patients who had died these data were ascertained for the 6 months preceding this event. Genetic analysis was detected for each patient. Sputum or bronchoaspirate swabs were obtained for each patient at regular clinical attendances (patients were reviewed at 3 months intervals) as well as twice during hospital admission. Chronic PA infection was defined as persistent presence of three PA positive cultures for at last 6 consecutive months [12]. Similarly, chronic pulmonary colonization by Bcc and other emergent pathogens was defined. Standard laboratory tests for PA antigens were regularly tested in our Centre but not applied to define chronic PA infection in this study.

The mean age of acquisition of chronic infection was registered. The yearly incidence of new chronic Gram-negative infection was computed. The prevalence of emergent pathogens infections was calculated as the total number of cases each year divided by the total number of patients during the observation period.

Co-infection was defined as the sputum culture positive for more than one organism. Patients were divided into four groups positive for PA, Bcc, PA and Bcc, SM.

Comparison between the effect of PA and Bcc on decline in lung function was performed during the observation period, as well as between Bcc and SM.

Multidrug-resistance was assessed according to Cystic Fibrosis Foundation for PA, extending it to all including clinically relevant non-fermentative Gram-negative strains [13].

PA multiresistant group was compared with its respective control group consisting of patients with non multiresistant PA infection.

### Processing of sputum samples, culture of organisms and phenotypic analysis

Natural or induced sputum samples, obtained from all patients involved during period of the study, were mixed with equal volumes of 1% dithiothreitol (Merck, Germany) before incubation at 37°C for 30 min. All specimens were examined microscopically and cultured. To isolate Bcc, specimens were incubated on the oxidation-fermentation base polymyxin B agar (Becton Dickinson) and BCSA agar (bioMérieux) at 37°C for up to 72 h. All isolates obtained from the samples were identified by the Phoenix system; the API 20 NE identification system (bioMerieux) was used when the identification with the Phoenix system was inaccurate. Preparation of suspensions, inoculations, incubation times, temperatures and interpretation of reactions accorded with the manufacturer's instructions for each system.

### Preparation of DNA from Bcc bacterial cultures

Liquid medium (25 ml Brain-Heart Broth, bioMérieux) in a test tube was inoculated with Bcc strains obtained from the solid culture medium. After incubation at 37°C with 120 shakes/min, at an optical density of 0.3–0.4 (absorbance at 600 nm) the cells were harvested by centrifugation at 4°C for 20 min at 3500 rpm. The pellet was resuspended in 4 ml of 50 mM D-glucose, 2 M Tris pH 7.5, 0.5 M EDTA pH 8. The cells were treated first with lysozyme (10 mg/ml) for 15 min at room temperature and then with 0.5 M EDTA, 10% SDS, followed by incubation with Proteinase K (0.2 mg/ml) at 55°C for 2 h. DNA was extracted with phenol, then with phenol and chloroform, and finally with chloroform only. Then it was precipitated with 0.1 volume Na-acetate and 2.5 volumes 99% ethanol. The pellet was rinsed three times with 70% ethanol, dried and then dissolved in 1 ml of TE buffer pH 7.8. The amount and purity of the bacterial genomic DNA were assessed by measuring the absorbances at 260 and 280 nm [14,15].

### Analysis for rec-A gene based identification

A DNA thermal cycler (PTC-100, MJ Research, Inc) was used. The Bcc *rec-A *gene (1.040 bp) was amplified using the primers BCR1 (tgaccgccgagaagagcaa) and BCR2 (ctcttcttcgtccatcgcctc), respectively, for the target 5' and 3' ends of the *rec-A *gene locus. PCR was performed in a total volume of 100 μl containing 0.5 γ/λ DNA, 25 mM MgCl_2_, 25 mM dNTPs, 5.80 pmol/μl BCR1, 10.40 pmol/μl BCR2 and 5 U/μl Taq-polymerase. After initial heating at 94°C for 1 min, 35 cycles of denaturation at 94°C for 30 sec, annealing at 59°C for 45 sec and extension at 72°C for1 min were performed. The final extension step was at 72°C for 5 min [14].

### RFLP analysis of Bcc rec-A gene

For RFLP analysis, the Bcc *rec-A *gene amplicons (up to 40 μl) were digested with *MnlI *(New England Biolabs, Inc) and *HaeIII *(New England Biolabs, Inc) restriction endonucleases in the appropriate buffers at 37°C for 2 h [14].

### Detection of PCR and RFLP products

The PCR-amplified products were analyzed by electrophoresis in 2% agarose gels. Restriction fragments were resolved in 3% high-resolution Agarose 1000 (Invitrogen, Inc) and the products were stained with ethidium bromide and observed under UV [14].

### Antimicrobial susceptibility testing method

To determine sensitivity to antimicrobial agents, a microbroth dilution assay using an automated Phoenix microdilution system was utilized. This system is based on serial dilutions of antibiotics and the MIC value is obtained from interpretative criteria for susceptibility breakpoints. Susceptibility criteria for the test method were in accordance with National Committee for Clinical Laboratory Standards Interpretative Criteria (Document M7-A4) [16].

### Statistical analysis

Before analysis, data were screened for gross deviance from normality. Since some groups of variables showed a skeweness of >1 with a small sample size, non-parametric tests for unpaired samples (Mann-Whitney test) were applied throughout the study. FEV1 values of PA group were compared with those belonging to Bcc group. FEV1 values of patients chronically infected with Bcc were compared to those of SM colonized patients. The effect of multidrug-resistant on FEV1 was analyzed within the PA group.

p value of less than 0.05 was considered statistically significant.

Statistical analysis was performed using SPSS version 12.0.

## Results

In the study time period, 3178 biological samples (sputum and/or bronchoaspirate swabs) were analyzed; 1917 Gram-negative strains were isolated.

During the first year of observation PA was isolated in 120 patients (40%) (range 1–13 years); in this group 81/120 patients (67%) were chronic infected over the time study period (mean age 8.7 years, range 1–19.9 years), with a mean FEV1 of 68% of predicted values for age and sex (range 15–122%). In 30/81 patients a multidrug-resistance was documented.

During the study period 21/300 patients (7%) were infected by Bcc; molecular analysis of *rec-A *gene showed that *B. cenocepacia *colonized 18 patient and three patients were colonized by *B. cepacia, B. stabilis *and *B. vietnamiensis *respectively, indicating that Bcc group most likely belongs to *B. cenocepacia*. 18/21 patients have multidrug-resistant Bcc chronic infection (mean age 10.8 years, range 7–16.6 years), with a mean FEV1 of 45% of predicted values for age and sex (range 15–80%).

AX was detected in 21/300 patients (7%); 7 (mean age 13.5 years, range 12–15.8 years) have chronic infection with a mean FEV1 of 53% of predicted values for age and sex (range 28–79%). SM was found in 33/300 (11%); 8 (mean age 10.6 years, range 10–15 years) were chronically infected with a mean FEV1 of 46% of predicted values for age and sex (range 26–82%). In order to correlate chronic PA and Bcc infection to lung function, patients chronically infected were divided into four groups when only one organism or co-infecting organisms were isolated.

Group 1: 65 CF patients with chronic PA infection were identified (mean age at chronic PA infection 8.9 years; range 1–19.9 years) with a mean FEV1 of 66.1% of predicted values for age and sex (range 19–122%)

Group 2: Bcc was isolated in 9 patients chronically infected (mean age at chronic Bcc infection 11 years; range 7–16 years) with a mean FEV1 of 46.0% of predicted values for age and sex (range 20–80%).

Group 3: 9 patients were chronically co-infected by PA and Bcc (mean age at chronic Bcc and PA infection 9.3 years; range 8–11.1 years) with a mean FEV1 of 46.6% of predicted values for age and sex (range 15–71%).

Group 4: 7 patients (mean age 10.6 years, range 10–15 years) were chronically infected with PA and SM and 1 patient only with SM, with a mean FEV1 of 46% of predicted values for age and sex (range 26–82%).

The distribution of PA and Bcc incidence and prevalence rate did not differ significantly during the study period. Annual prevalence rate during each year of observation ranged from 11% in 2002 to 17% in 2004 for SM.

Patients with Bcc chronic infection (Group 2 and Group 3) showed FEV1 mean value significantly lower than that shown by patients with only PA chronic infection (Group 1) (Group 1 vs Group 2, p = 0.03; Group 1 vs Group 3, p = 0.002). Co-infection with PA in Bcc patients did not lead to significant variation of FEV1 mean values (Group 2 vs Group 3, p = 0.9). Infection with SM did not effect lung function of Bcc colonized patients.

In patients chronically PA colonized a significant decrease of FEV1 mean value was found in patients (n = 30) with PA multidrug-resistance (p < 0.001).

Other Gram-negative bacteria such as *Comamonas acidovorans, Comamonas testosteroni, Acinetobacter calcoaceticus *and *Chryseobacterium indologenes *were occasionally isolated with the other organisms.

In table 1 total number of isolates, number and percentage of multiresistant strains for each type of bacteria are reported.

**Table 1:** Distribution (number and percentage) of multidrug-resistant strains during the study period

	Total strains	Total strains	Multidrug-resistant strains	
	No	%	No	%
P.aeruginosa	1187	61.9	115	9.6
S.maltophilia	277	14.4	76	27.4
A.xylosoxidans	166	8.6	62	37.3
B. cepacia	14	0.7	14	100
B.cenocepacia	266	13.8	187	70.3
B.stabilis	4	0.2	4	100
B.vietnamiensis	3	0.1	2	66.7
				
	*1917*		*460*	*23.9*

Drug-multiresistance was found in 30 chronically PA infected patients. Multiresistant Bcc strains were found in 18 patients, all chronically infected.

Susceptibility studies of multidrug-resistant pathogens over the study period are summarized in Table 2. Beta-lactam antibiotics showed moderate activity in vitro against multiresistant bacteria of all species: only ceftazidime seems to be the most efficacious antibiotic against these types of bacteria (45.4%, 16.6%, 55.7% respectively against Bcc, PA and AX) Therefore, ceftazidime was the most active antibiotic against Bcc, while imipenem was the most active in vitro against AX. Aminoglycosides are not very active against the bacterial strains isolated and quinolones are relatively inactive against multiresistant strains.

**Table 2:** Percentage of Gram-negative isolated strains resistant to antibiotics tested.

**Antibiotics**																			
	**AMK**	**AMP**	**ATM**	**FEP**	**CTX**	**CAZ**	**CIP**	**CHL**	**COL**	**GEN**	**IPM**	**LVX**	**MEM**	**NET**	**PIP**	**TZP**	**RIF**	**TET**	**SXT**
*Bcc*	98.46	100	81.82	81.82	86.62	45.45	89.06	87.50	100	98.46	91.30	83.33	65.63	96.97	65.15	51.52	93.94	100	76.92
*SM*	94,23	100	98.11	89.52	94.29	83.02	94.34	51.92	40	96.23	100	66.98	98.08	83.33	91.35	89.52	80	94.23	44.76
*PA*	56.16	100	20.55	15.71	78.08	16.67	44.44	89.39	4.88	61.64	38.46	49.32	38.36	46.30	43.06	20.55	96.23	96.49	100
*AX*	80	100	92.86	87.14	92.86	55.71	79.71	54.41	76.47	88.57	15.56	60	31.88	89.47	25.71	14.71	93.75	72.31	47.14

AMK = *Amikacin*; AMP = *Ampicillin*; ATM = *Aztreonam*; FEP= *Cefepime*; CTX= *Cefotaxime*; CAZ = *Ceftazidime*; CIP = *Ciprofloxacin*; CHL = *Chloramphenicol*; COL = *Colistin*; GEN = *Gentamicin*; IPM = *Imipenem*; LVX = *Levofloxacin*; MEM = *Meropenem*; NET = *Netilmicin*; PIP = *Piperacillin*; TZP = *Piperacillin-tazobactam*; RIF = *Rifampin*; TET = *Tetracycline*; SXT = *Trimethoprim-sulfamethoxazole*.

## Discussion

This observational study examines the most prevalent pathogens in a CF cohort of a single care Centre. PA is identified more frequently in keeping with previous findings [17].

Our study has confirmed that patients colonized with Bcc and PA multidrug-resistant strains have a more rapid lung decline with an associated increased number of hospital admission (data not shown) [18]. With improved survival, new pathogens have emerged in the CF lung, particularly including SM and AX. The importance of this colonization in CF patients is still unclear. Only a few previously case-control studies have been performed which found no definitive evidence with a worse prognosis. Treatment of chronic infection is controversial and not so successful from a bacteriological point of view. However, in many CF centres it is generally believed that the progressive tissue damage due to chronic inflammation caused by opportunistic pathogens requires an early and promptly regular treatment employing large dosage of antibiotics. This approach has been generally shown to be successful compared with courses of antibiotics administered only for acute exacerbations. For this reason the heavy antibiotic exposure in CF patients leads to multidrug-resistance due to different mechanisms [19].

Our observational study reports the experience of incidence and prevalence of Gram-negative pathogens in our CF care Centre that applies a long term intensive treatment for chronic infection.

In our Centre overall percentage was 40%, 7% and 11%, respectively for PA, Bcc and SM in 2002, according to data worldwide [17] with no marked increase of prevalence over the time study period, except for SM which has shown an increasing prevalence from 11% to 17% during the three years.

*B. cenocepacia *was the most prevalent genomovar of Bcc recovered in our population. Moreover, patients were colonized only by single types of genomovar. Our data confirm that Bcc affects the clinical outcome of CF patients above that PA, the most prevalent pathogen for this group of patients [18]. There was a greater overall number of deaths for patients with PA multidrug-resistant infection in comparison with matched patients with PA infection (36% vs 5.8%). The overall number of deaths in all multidrug-resistant Bcc patients was 33.3%. The factors influencing the clinical course of a patient following acquisition of chronic infection are clearly complex and probably influenced by multiple factors, including host response to pathogens. Probably multidrug-resistance can influence prognosis and it is confirmed by our results that show effect of PA multiresistance on deterioration of lung function compared to matched patients with PA infection. Our analysis indicates that many multiresistant strains are found in our Unit: of the strains isolated, 23.9% are multiresistant, as showed in table 1. As previously reported, our study also suggests that Bcc as well as SM and AX, is resistant to many classes of antibiotics [20-22].

For this reason it could be a good clinical practice to investigate antibiotic susceptibility and synergy testing of multidrug-resistant pathogens with conventional antibiotics. A multiple combination bacterial testing is recommended for testing tobramycin and beta-lactams and/or third-generation cephalosporins, according to conventional therapy.

Regular assessment of airway colonization is one of the basic guidelines in clinical follow-up of these patients. Identification of Gram-negative pathogens from the respiratory tracts is critical not only for individual care but also for the CF community. This is particularly true for such bacteria as *B. cenocepacia *and other Bcc species because they are highly transmissible and have a clinically significant impact on mortality [23,24,13]. Molecular diagnostic probes based on the PCR provide a rapid and frequently highly discriminatory means of identifying infectious organisms. Use of a PCR-RFLP method to establish the bacterial genomovar of Bcc is very important; several studies have investigated potential differences in the clinical impacts of different genomovars [22,25]. As described above, *B. cenocepacia *appears more likely than other genomovars to be spread among patients and to be associated with outbreaks [26,27]. The small number of our data does not consent to confirm these results.

It is not clear whether new pathogens could compromise the clinical status of CF patients. Large-scale clinical trials are needed to assess their potential pathogenic role in accelerating the decline in lung function [28-30].

The pathogenicity of SM and AX in CF is not yet established, although an association of these microorganisms with pulmonary exacerbation has been reported. SM chronic infection in our patients does not further influence clinical outcomes.

This study shows that there is no treatment of choice for multidrug-resistant organisms. Therefore, synergistic antibiotic combinations may be useful for identifying the best therapeutic action against multiresistant strains.

## Conclusion

Our data emphasize the crucial role of microbiological methods in defining possible therapeutic strategies that may help to guide antibiotic therapy regimes in CF patients. The short follow-up period and the small number of some groups of patients infected with individual strains don't allow to define survival analysis and to correlate microbiological data to clinical outcomes between groups of patients. Further longitudinal prospective studies are ongoing.

## Competing interests

The author(s) declare that they have no competing interests.

## Authors' contributions

Lambiase A.

Collected data relating to the study and drafted the paper

Raia V.

Collected data relating to patients involved in the study

Del Pezzo M.

Coordinated the analysis of clinical specimens

Sepe A.

Partecipated in the study design and coordination

Carnovale V.

Partecipated in the study design and coordination

Rossano F.

Conceived of the study and partecipad in its design and coordination

All authors read and approved the final manuscript

## Pre-publication history

The pre-publication history for this paper can be accessed here:


